# Novel Technologies in Breast Imaging: A Scoping Review

**DOI:** 10.7759/cureus.44061

**Published:** 2023-08-24

**Authors:** Nicole F Grigoryants, Sarah Sass, Julia Alexander

**Affiliations:** 1 Medicine, Alabama College of Osteopathic Medicine, Mobile, USA; 2 Medicine, Alabama College of Osteopathic Medicine, Dothan, USA; 3 Diagnostic Radiology, Alabama College of Osteopathic Medicine, Dothan, USA

**Keywords:** contrast-enhanced imaging, breast cancer imaging, diffusion-weighted imaging (dwi), digital tomo-synthesis, screening of breast cancer, primary breast cancer prevention, proton magnetic resonance spectroscopy

## Abstract

Breast cancer is one of the leading causes of death in the United States and can cause considerable suffering for not only the patient but their families as well. The current mainstay of screening is mammography, although this screening modality has its drawbacks. Multiple technologies have been recently explored in hopes of increasing breast cancer detection rates and decreasing false positive rates. Overall, improving breast cancer screening techniques has the potential to decrease cost, patient anxiety, and the use of unnecessary procedures. This review discusses multiple modalities including digital breast tomosynthesis, contrast-enhanced dual-energy digital mammography (CE DE DM), MRI with diffusion-weighted sequences and proton magnetic resonance spectroscopy. This paper was written with the objective of synthesizing information across several databases to provide clinicians with a more accessible tool to understand the underlying concepts behind these imaging modalities, as well as present reviewed data which highlights the benefits and drawbacks of these breast cancer-detecting techniques.

## Introduction and background

Breast cancer is one of the most common and deadliest cancers for women in the US, with 297,790 predicted new cases and 43,170 estimated deaths in 2023 by the National Cancer Institute [1]. Innovations in screening modalities have been consistently explored in the hopes of developing technology that optimizes sensitivity and specificity while decreasing patient discomfort and radiation exposure. Although mammography remains the mainstay of breast cancer screening, it has been found to be variable in its sensitivity, ranging from 37% to 71% but with about 95% specificity [2,3]. MRI is considered the most sensitive technology for breast imaging with a sensitivity found anywhere from 70% to 100%. However, MRI is generally utilized in high-risk cases and may be considered relatively inaccessible due to its “high cost and limited availability,” according to Sung et al. [4]. These shortcomings result in greater resource demands, false-positive results (potentially leading to increased patient anxiety), and unnecessary confirmatory procedures such as biopsies [3,5].

Standard breast cancer screening protocols may recommend imaging to begin in women as young as 30 years of age in high-risk categories, but they generally suggest biennial mammograms for women ages 40-49 and annual mammograms for women ages 50-75 [6]. Currently, two forms of mammography exist: film screen and digital. Film screen mammography is anywhere from 1.5 to 4.0 times less expensive than digital mammography, has been found to result in fewer false positives, and requires less-costly monitors for viewing images. In contrast, digital mammography, which comprises over 95% of the mammography systems used today, proves advantageous over film-based systems as it delivers a lower average radiation dose, provides greater spatial and contrast resolution, and allows for post-processed readings [7,8].

**Figure 1:**
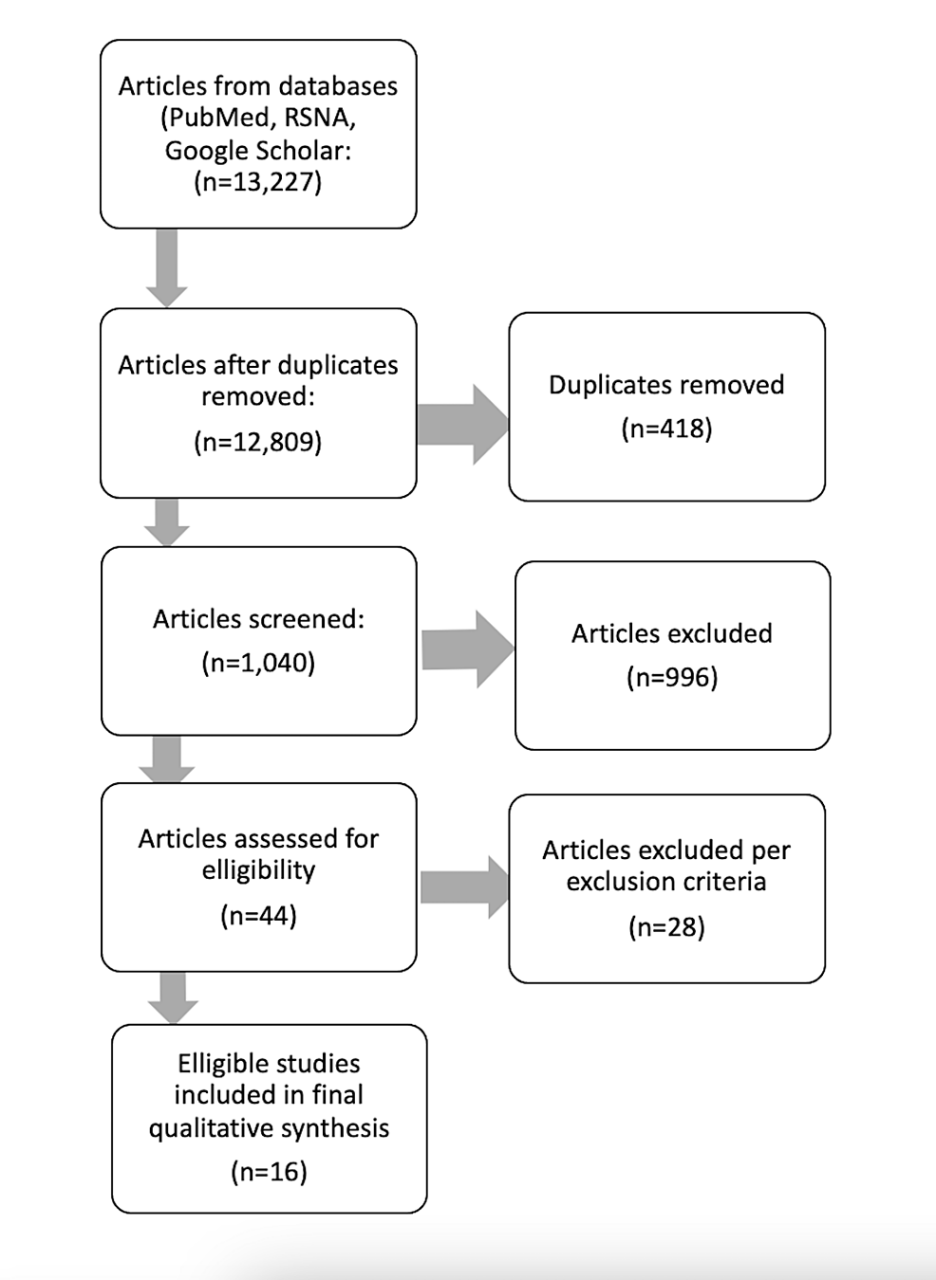
Flow diagram documenting article obtainment, selection, and review process

This study was a scoping review of references that investigated the efficacy of imaging modalities that have become increasingly popular for establishing breast cancer diagnoses. Article databases, clinical information tools, and medical journals from which referenced studies were derived include PubMed, UpToDate, Google Scholar, and the Radiological Society of North America (RSNA). The most common keywords searched, yielding relevant studies, included phrases such as “new breast imaging modalities,” "breast cancer imaging techniques," and “emerging breast cancer screening technology.” Two independent reviewers surveyed all of the above databases. For inclusion, studies had to have discussed either the scientific principles or data supporting the efficacy of any of the following imaging modalities: digital or film mammography, standard magnetic resonance imaging (MRI), digital breast tomosynthesis (DBT), contrast-enhanced dual-energy digital mammography (CE DE DM), MRI with diffusion-weighted imaging (DWI), or proton magnetic resonance spectroscopy (^1^H-MRS). The article deduction flow chart is depicted in Figure 1. 

Researchers also applied a language qualification whereby studies available exclusively in English in the article databases were included. However, it is worth noting that some studies may have been originally written in other languages and subsequently translated to English upon submission to these databases. Articles published or edited more than 15 years before the inception of this report on July 12, 2022, were excluded. A total of 16 sources were deemed eligible for review after duplicate removal and implementation of exclusion and inclusion criteria. Additionally, several searches from PubMed and RSNA yielded over 10,000 search results. In order to reliably refine these results, we concluded each search when 20 or more consecutive articles were found not pertinent to our study.

The objective of this study was twofold: 1) to review and compare recently developed breast imaging modalities and 2) to consolidate this information from a vast study pool. By satisfying this objective, researchers hope to provide clinicians with a comprehensive source that gives foresight on the trajectory of breast cancer screening technologies. This review will discuss four relatively new breast imaging technologies: digital breast tomosynthesis (DBT), contrast-enhanced dual-energy digital mammography (CE DE DM), MRI with DWI, and proton magnetic resonance spectroscopy (^1^H-MRS). The aim of our paper is not to challenge current breast cancer screening guidelines but rather to discuss new imaging modalities that are being investigated to improve screening outcomes. Doing so will improve patient mortality and reduce false-positive rates, which will decrease the stress placed on the United States healthcare system by reducing the number of unnecessary biopsies.

## Review

Digital breast tomosynthesis

One new imaging technique is DBT. This screening modality was developed to be utilized in conjunction with digital mammography to increase its sensitivity and specificity and decrease the rate of false positives. DBT utilizes a moving x-ray and digital detector to achieve a three-dimensional mammography image. According to Elmore and Lee, there was an increase of 1.6 cancers per 1000 detected when tomosynthesis was used as well as a reduced recall rate [3]. One of the drawbacks of this imaging technique is the risk of increased radiation. When DBT was performed with full-field digital mammography, the cancer detection rate was increased by 89%, but the radiation dose nearly doubled and could be greater with more dense breasts [8].

**Figure 2:**
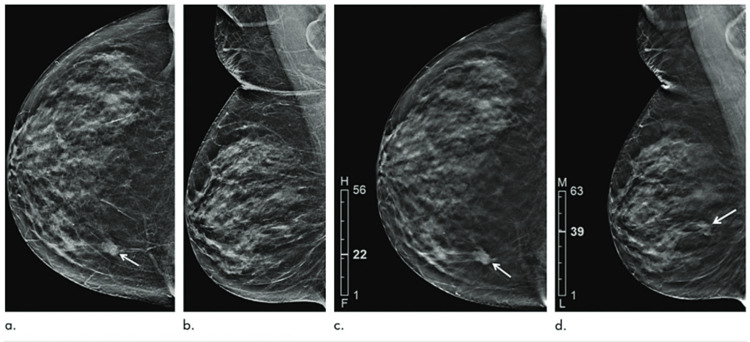
A 78-year-old patient presented with a palpable mass in the right breast, with images obtained from digital mammography and DBT revealing invasive ductal carcinoma Panels A and B show images from digital mammography in craniocaudal (CC) and mediolateral oblique (MLO) views, respectively. The mass is better visualized using the CC view, and Panel C shows a DBT CC stack that localizes the lesion (arrow) further. Using this information, Panel D reveals the mass (arrow) in the MLO view as well. *Source*: Chong A, Weinstein S P, McDonald E S, et al. [9], with permission from the Radiological Society of North America.

In a study by Nakajima et al., 186 breasts were evaluated using full-field digital mammography (FFDM) with DBT and two-dimensional synthetic mammography (SM) with DBT. The sensitivity and specificity were equivalent between the two groups, but higher radiation was needed for FFDM/DBT versus SM/DBT [9]. This shows promise in the use of tomosynthesis as it increases the detection rate of breast cancers and decreases recall rates. DBT can more readily demonstrate lesions as compared to the corresponding synthetic view. Figure 2 demonstrates the difference between standard digital mammography and DBT in the detection of suspicious lesions.

The use of digital breast tomosynthesis has been shown in various studies such as the study by Venkatamaran et al. and Nakajima et al. to be superior to standard full-field digital mammography as a breast cancer screening method [8,9]. As DBT acts as a way to take thousands of images of the same point in a breast from different angles along an arc, it provides a more detailed image of the breast that helps clarify any suspicious areas seen on mammogram since the superimposed benign tissues from a two-dimensional perspective may appear more clinically questionable [10]. Though not much varies significantly regarding patient experience when obtaining this scan compared to a mammogram, the concern of increased radiation must be considered when assessing the appropriateness of DBT for a patient [8]. However, increased sensitivity and specificity in cancer detection rates as compared to mammography alone support DBT as a preferred method of cancer screening, particularly in patients with dense breasts.

Contrast-enhanced dual-energy digital mammography (CE DE DM)

**Figure 3:**
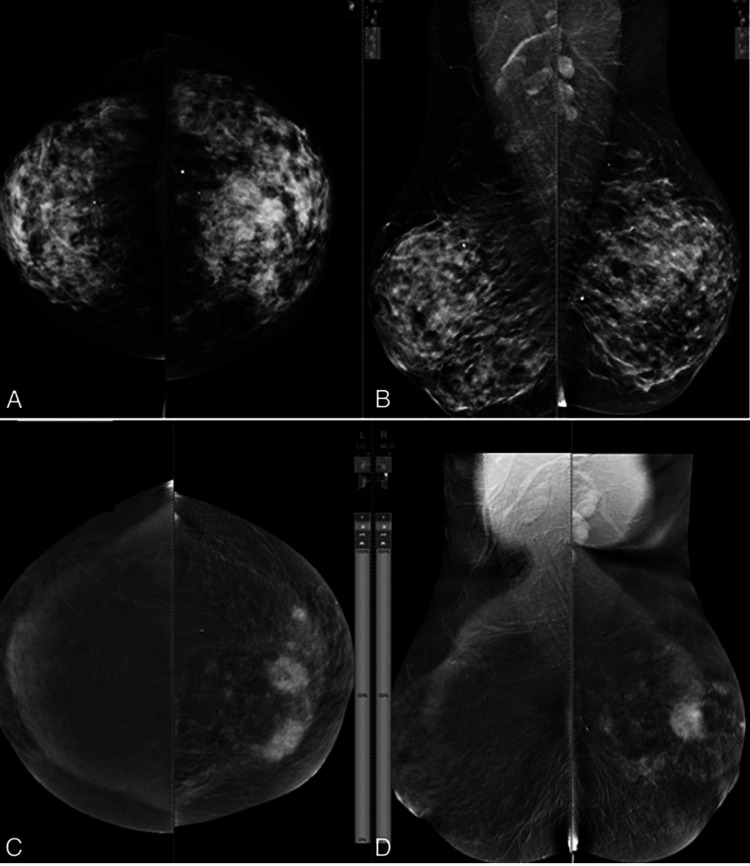
Comparing standard mammography to contrast-enhanced dual-energy digital mammography in the imaging of invasive lobular carcinoma Invasive lobular carcinoma of the left breast in a 55-year-old patient. Panels A and B are mammographic images in which the borders of a mass in the left breast are not well demarcated and calcifications are less discernible. The images in panels C and D, using CE DE DM, suggest multifocal breast cancer, as lesions are at 10 o'clock and 12 o'clock. Additionally, the borders of the upper outer and inner calcifications are more clearly visualized with the use of this technology. The use of CE DE DM suggests multicentric breast cancer, which was further confirmed by mastectomy. *Source: *Moustafa AFI, Kamal EF, Hassan MM, Sakr M, Gomaa M [13] with permission of image used under Creative Commons License: CC BY-NC-ND 4.0.

Contrast-enhanced, dual-energy digital mammography (CE DE DM) is an emerging breast imaging technology wherein a contrast-agent-based image obtained at low energy is then overlaid onto a subtracted image [11]. This allows for both an “anatomical and functional” image of the breast: highlighting calcifications from the low-energy component and neovascularization depicting angiogenesis from the subtracted image [12]. While CE DE DM was introduced in 2003, it was not approved for clinical imaging by the Food and Drug Administration until 2011 [12]. In recent years, this imaging modality has been investigated as an alternative breast cancer screening technique to DBT, rather than as an adjunct used in inconclusive studies. Studies support that CE DE DM can show calcifications otherwise undetected in 2D digital mammography. Additionally, this imaging technique has been theorized to succeed in its detection upon screening of invasive lobular carcinoma (ILC), which is known to be a difficult breast cancer as it may not form a discernible palpable mass; however, there have yet to be any studies to confirm this [12]. As demonstrated in Figure 3, the use of CE DE DM for the diagnosis of ILC may be more accurate than compared to standard mammography. Research has also shown that CE DE DM is especially useful for breast cancer detection in dense breasts when used in conjunction with mammography [2].

Though CE DE DM has also been found to have other numerous notable advantages such as greater diagnostic sensitivity, fewer false positive results, and higher negative predictive value than standard 2D digital mammography, its specificity has been found to be significantly variable compared to that of the current mainstay of breast cancer screening [14]. It has been suspected that multiple foci of contrast-enhanced tissues at different depths may result in ambiguity in lesion location and morphology [15]. It should also be noted that the use of contrast is not effective independent of time and is not an ideal choice in all patients, specifically in cases of impaired renal function (pathological or drug-induced) or allergy.

MRI with diffusion-weighted imaging (DWI)

**Figure 4:**
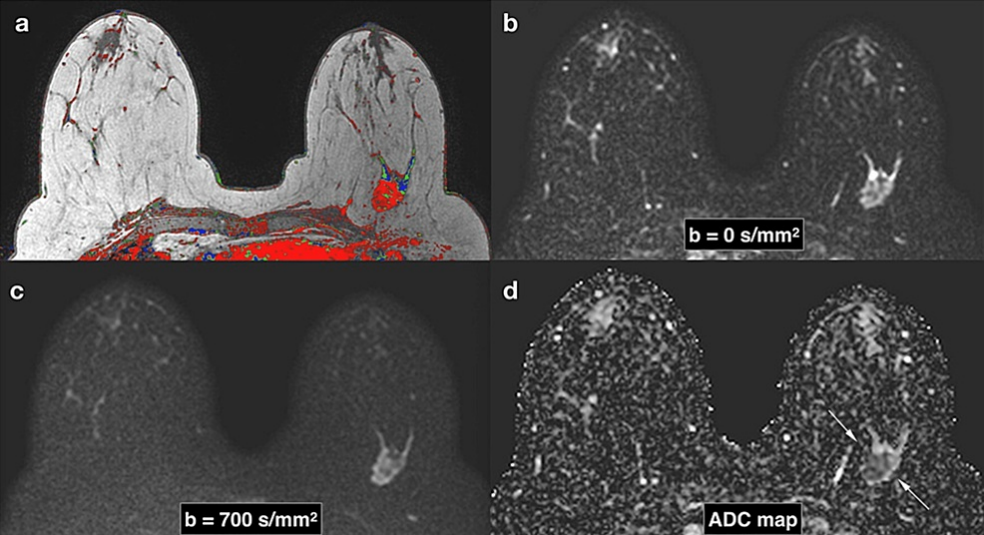
Triple-negative subtype left breast cancer shown on MRI with diffusion-weighted sequencing with corresponding ADC map Panel A shows a dynamic contrast-enhanced MRI post-contrast highlighting a posterior left breast cancer. Shown in Panel B is the MRI with diffusion-weighted sequencing at b=0 s/mm2, where B reflects the degree of diffusion weighting applied. Panel C shows the same breasts at b=700 s/mm2, which does improve the emphasis placed on the suspicious lesion. Both Panels B and C demonstrate that malignant lesions are hyperintense on DWI. Panel D corresponds to the ADC map in which the left breast cancer appears hypointense. *Source*: Camps-Herrero J. [17] with permission of image used under Creative Commons License: CC BY 4.0.

MRI with DWI processes molecular motion patterns along a magnetic field gradient in tissue to produce an interpretive image of the breast. Since cancer lesions generate abnormal water movement patterns, this motion is quantified so that tumors may be visualized [16]. This quantification of water diffusion, known as diffusivity, is calculated and reported as an apparent diffusion coefficient (ADC), spatially represented on what is called an ADC map. Malignant breast cancers have lower ADC values as water movement is restricted, allowing for distinctions to be made between normal breast tissue, benign tumors, and invasive cancers [16]. Though studies have been limited, there has been evidence showing that DWI can display distinctions between benign and malignant lesions, which are otherwise unable to be visualized using mammography or tomosynthesis. Emerging data from Slanetz et al. shows that DWI may be able to differentiate between benign and malignant lesions with “equal sensitivity and greater specificity than MRI,” and in one study resulted in a 34.5% reduction in unnecessary and invasive MRI-guided biopsies without compromising negative predictive value [16]. The utilization of MRI with diffusion-weighted sequencing has been estimated to have the potential to reduce costs by over 30% through decreasing unnecessary procedures [16]. Figure 4 exemplifies the strengths of MRI with DWI with a corresponding ADC map in the detection of suspicious lesions.

Overall, the strengths of MRI with DWI are clear; however, its disadvantages preclude from using DWI as a primary breast cancer screening modality as it exists today. The morphological and microstructural elements of benign and malignant breast tumors overlap to a significant extent, which translates to similar overlap in ADC values. Therefore, DWI has “limited clinical value” without a clear and consistent ADC cutoff value aside from when used to support previously determined diagnoses [12]. Finally, DWI cannot be used alone for asymptomatic patients or suspicious lesions detected by mammography [16]. Relevant studies found for this review recommend the use of DWI not in place of mammography or MRI but rather as a confirmatory adjunct. The use of DWI as a supplement to MRI (more specifically, MRI-guided biopsy) is suggested to be particularly useful during the procedure, potentially aiding in the location of certain tumors or localization of the most aggressive points within a tumor. Because this technique serves as a way to be able to map the cancer microenvironment, trends within ADC values can suggest tumor receptiveness to certain hormone markers over others.

Proton magnetic resonance spectroscopy (^1^H-MRS)

**Figure 5:**
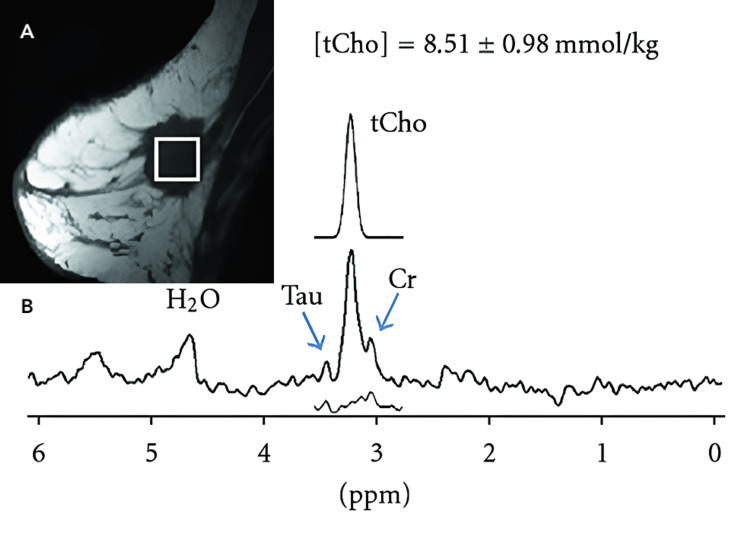
MRI and MRS showing a mixed invasive ductal and lobular carcinoma The image from a sagittal breast MRI in the top left corner shows a suspicious 2.8 cm lesion (Panel A), which was confirmed by surgical resection and pathological analysis as a mixed invasive ductal and lobular carcinoma. A corresponding ^1^H-MRS (Panel B) shows a water-fat suppressed spectrum with a choline residue peak (labeled as tCho) at 3.2 ppm, a taurine (Tau) peak at 3.45 ppm (left blue arrow), and a creatinine (Cr) peak at 3.02 ppm (right blue arrow). An estimated Gaussian fit curve is shown above tCho with a measurement of 8.51 ± 0.98 mmol/kg. *Source*: Baek, H.-M. [18] with permission of image used under Creative Common License: CC BY 3.0.

Proton magnetic resonance spectroscopy (^1^H-MRS), like diffusion-weighted imaging, is a noninvasive technology with no radiation that has recently been investigated in the context of breast cancer diagnosis. In vivo studies showed that ^1^H-MRS can provide a breakdown of the chemical composition of a tissue [5]. This means that the technique is able to distinguish between benign and malignant lesions by detecting choline levels, the metabolites of which are increased in invasive cancers due to an increase in cell turnover and metabolic process rates [16]. The choline metabolite phosphocholine is a reliable chemical to monitor as it displays as a single spectroscopic peak. Previous studies summarized by Geraghty et al. found that the use of ^1^H-MRS gave results with sensitivities ranging from 70% to 100% and specificities from 67% to 100%. This study also found a positive predictive value of 88% [5]. Figure 5 demonstrates the use of ^1^H-MRS with post-contrast MRI in the context of an invasive ductal carcinoma.

As the name implies, ^1^H-MRS must be paired alongside an imaging modality in order to localize suspicious breast tissue: this imaging counterpart has almost exclusively been (often contrast-enhanced) MRI in existing studies, and any variation from this would theoretically pose a redundancy. Another evident caveat to using ^1^H-MRS is that a significant source of false negatives stems from the fact that not all cancers have high levels of choline and may even be completely choline-negative in general. According to Geraghty et al., results supported that less aggressive cancers were not as likely to have consistently high spectroscopic peaks [5]. However, this shortcoming may be minimized by cross-referencing the spectroscopy results of suspicious lesions with a value called k21. This value numerically represents “the exchange rate of the used contrast agent between the intra- and extravascular spaces” [5]. When a choline peak is present, its height generally corresponds with the magnitude of the k21 value.

## Conclusions

Overall, this scoping review aimed to investigate the benefits and drawbacks of four different breast cancer imaging methods: DBT, CE DE DM, MRI with DWI, and ^1^H-MRS. These imaging modalities have become more heavily investigated in recent years and therefore warrant discussion among medical professionals beyond the specialty of radiology. Limitations of this study include the time constraints and expertise of the reviewers involved. This study found that digital breast tomosynthesis was often cited as an imaging modality that increased cancer detection rates and negative predictive value. Unlike DBT, an advantage of contrast-enhanced dual-energy digital mammography is its high sensitivity in detecting underlying neoplasms in all breast densities as well as increasing the likelihood of detecting microcalcifications unnoticed on mammography. Despite these advantages, the summation of contrast-enhanced areas may lead to ambiguity in these scans. Finally, this imaging modality uses contrast, which may not always be appropriate for certain patient populations. DWI provides an immediate assessment of ADC values, which may be able to distinguish between benign and malignant. However, MRI with DWI currently lacks sufficient support for clear ADC value thresholds, which may limit its use in cases where a lesion’s ADC is equivocal. Our final novel technique discussed, proton magnetic resonance spectroscopy, is an imaging modality that is noninvasive and involves no radiation but has been found to have weaker specificity than either MRI or mammography.

The investigation of novel breast imaging technologies is an important area of study for its medical and social implications. Breast cancer screening is a source of anxiety for many patients, and any advancement toward improving monetary cost, scan-to-result time, and accuracy will decrease patient apprehension. By increasing accuracy and precision, we can expect lower stress placed on the healthcare system due to minimizing unnecessary procedures and increasing efficiency in resource distribution. While it is evident that no screening method has been developed yet that is more reliable or practical than mammography, innovations in this field are noteworthy enough to study for the prospect of more successfully managing all breast cancer cases.
